# Immunogenicity and Protective Capacity of a Virosomal Respiratory Syncytial Virus Vaccine Adjuvanted with Monophosphoryl Lipid A in Mice

**DOI:** 10.1371/journal.pone.0036812

**Published:** 2012-05-09

**Authors:** Tobias Kamphuis, Tjarko Meijerhof, Toon Stegmann, Julia Lederhofer, Jan Wilschut, Aalzen de Haan

**Affiliations:** 1 Department of Medical Microbiology, Molecular Virology Section, University Medical Center Groningen, University of Groningen, Groningen, The Netherlands; 2 Mymetics BV, Leiden, The Netherlands; University of Iowa, United States of America

## Abstract

Respiratory Syncytial Virus (RSV) is a major cause of viral brochiolitis in infants and young children and is also a significant problem in elderly and immuno-compromised adults. To date there is no efficacious and safe RSV vaccine, partially because of the outcome of a clinical trial in the 1960s with a formalin-inactivated RSV vaccine (FI-RSV). This vaccine caused enhanced respiratory disease upon exposure to the live virus, leading to increased morbidity and the death of two children. Subsequent analyses of this incident showed that FI-RSV induces a Th2-skewed immune response together with poorly neutralizing antibodies. As a new approach, we used reconstituted RSV viral envelopes, i.e. virosomes, with incorporated monophosphoryl lipid A (MPLA) adjuvant to enhance immunogenicity and to skew the immune response towards a Th1 phenotype. Incorporation of MPLA stimulated the overall immunogenicity of the virosomes compared to non-adjuvanted virosomes in mice. Intramuscular administration of the vaccine led to the induction of RSV-specific IgG2a levels similar to those induced by inoculation of the animals with live RSV. These antibodies were able to neutralize RSV *in vitro*. Furthermore, MPLA-adjuvanted RSV virosomes induced high amounts of IFNγ and low amounts of IL5 in both spleens and lungs of immunized and subsequently challenged animals, compared to levels of these cytokines in animals vaccinated with FI-RSV, indicating a Th1-skewed response. Mice vaccinated with RSV-MPLA virosomes were protected from live RSV challenge, clearing the inoculated virus without showing signs of lung pathology. Taken together, these data demonstrate that RSV-MPLA virosomes represent a safe and efficacious vaccine candidate which warrants further evaluation.

## Introduction

Respiratory Syncytial Virus (RSV) is a major cause of viral brochiolitis in infants and young children and is also a significant problem in elderly and immuno-compromised adults. According to the WHO, annually 64 million people are infected with RSV, and 160,000 people die from the infection around the world [1]. It is estimated that, each year, RSV leads to 3.4 million hospitalizations of children [2]. By the age of two, nearly all children have been infected with RSV. However, natural infection does not evoke long-lasting immunity, which causes people to undergo multiple RSV infections throughout their lives. In healthy adults, RSV infection will manifest itself like a common cold, which is generally cleared within two weeks. When, at old age, the immune system weakens, RSV infections become more severe, leading to, for example, approximately 10,000 deaths in nursing homes in the US each year [3], [4]. Current treatment of RSV infection in high-risk infants consists of prophylactic administration of the monoclonal antibody Palivizumab [5]. However, the high costs of monoclonal antibody therapy and the limited duration of efficacy of this treatment warrant the development of an RSV vaccine [6], [7] In elderly, treatment is mainly supportive and consists of administration of fluids, oxygen and antipyretics [8]. Aerosolized Ribavirin is registered for use in some infant groups however, no significant effect has been reported in the elderly [8].

Even though the need for an RSV vaccine has been recognized for over 60 years, there is currently no licensed RSV vaccine available. This is, in part, due to the disastrous outcome of a clinical trial in the 1960s, which evaluated a formalin-inactivated, alum-adjuvanted, RSV (FI-RSV) vaccine candidate [9]–[12]. In this trial, children who received the vaccine developed RSV-specific antibodies, but these proved to be poorly virus-neutralizing [13], [14]. Instead of preventing infection, vaccination resulted in enhanced respiratory disease (ERD) upon infection with the live virus, leading to a 16-fold increase in hospitalization and even to the death of two children in the vaccinated group [15]. After this trial, many studies have been performed to elucidate the mechanisms causing ERD upon vaccination with FI-RSV and subsequent exposure to live virus. Studies in mice, for example, showed that a Th2-like immune response accompanied by influx of eosinophils into the lungs plays a major role in ERD [16]. Another study in mice has shown that, although FI-RSV does elicit RSV-specific antibodies, these have a limited affinity for neutralizing epitopes on the RSV fusion protein due to lack of affinity maturation [17]. Therefore, a future RSV vaccine should induce a Th1-skewed response together with high levels of strongly neutralizing antibodies.

A promising approach towards the development of vaccines that both skew the immune response to a Th1-type reaction and induce high-affinity antibodies is to include Toll-Like Receptor (TLR) ligands in the vaccine [18]. TLRs recognize Pathogen-Associated Molecular Patterns (PAMPs) from bacteria and viruses and subsequently signal through adaptor molecules such as MyD88 and TRIF to induce the production of inflammatory cytokines and type-I interferons [19]. Activation of TLR4, for example, leads to production of high amounts of IL12 and IFNγ resulting in a Th1-skewed immune response [20]. Importantly, a recent study showed that a UV-inactivated RSV virus, which by itself induces poorly neutralizing antibodies, will give rise to high-affinity and strongly neutralizing antibodies when supplemented with TLR ligands [17]. Using a similar approach, we recently showed that the incorporation of the TLR2 ligand P3CSK4 in an experimental virosomal RSV vaccine promotes the capacity of the vaccine to induce Th1-type cellular responses together with the induction of protective antibodies in mice and cotton rats [21]. Thus, the combination of an RSV vaccine, such as RSV virosomes, with a TLR ligand improves both the immunogenicity and the safety of the vaccine.

Another promising TLR ligand candidate to be used as an adjuvant in an RSV vaccine is the TLR4 ligand monophosphoryl lipid A (MPLA) [22]. MPLA is a detoxified derivative of bacterial lipopolysaccharide (LPS) [23]. Like LPS, MPLA also signals through TLR4. However, where TLR4 activation by LPS induces signaling through adaptor molecule MyD88, activation by MPLA leads to TRIF-mediated signaling, resulting in enhanced type I IFN production and reduced production of inflammatory cytokines compared to MyD88-mediated signaling [17], [24], [25]. MPLA stimulates the production of IFNγ by antigen-specific CD4+ T-cells indicating a Th1-skewed response [22], [26]. While the TLR2 ligand P3CSK4, which we used in our previous study [21], has been associated with a balanced Th1/Th2-type immune response, MPLA is thus known to induce a significantly Th1-skewed immune response [27]. Furthermore, an MPLA derivative with similar immune-potentiating properties as native MPLA has been evaluated in extensive clinical trials and has shown good efficacy combined with an acceptable safety profile for use in humans when co-administered with a variety of antigens [28]. For these reasons, MPLA is the only TLR ligand which is currently being used as an adjuvant in a number of licensed vaccines [29], [30]. Importantly, the addition of MPLA to FI-RSV suppressed the expression of RSV ERD associated cytokines in the lungs of cotton rats [31]. Furthermore, it has been shown that addition of MPLA to FI-RSV promotes the immunogenicity of the vaccine and ameliorates lung pathology after challenge [32]. Thus, the favorable Th1-inducing properties of MPLA, compared to P3CSK4, combined with the available data on the inhibitory effects of this TLR ligand on the development of RSV ERD and its acceptable safety profile in humans, led us to explore MPLA as a possible adjuvant in our RSV virosomal vaccine.

We exploited the lipophilic properties of MPLA to incorporate it in the virosomal membrane during the reconstitution process. These virosomes were analyzed for their immunostimulating properties and immunogenicity both *in vitro* and *in vivo* and for their capacity to induce protection against infection with live RSV. Our data show that incorporation of MPLA in RSV virosomes increases their immunostimulatory capacity *in vitro,* as evidenced by increased human TLR4-mediated NF-κB activation and upregulation of costimulatory molecules in mouse dendritic cells. *In vivo*, incorporation of MPLA in RSV virosomes stimulated RSV-specific IgG antibody levels, with increased IgG2a antibody production and increased levels of virus neutralizing antibodies compared to non-adjuvanted RSV virosomes. Also, RSV-MPLA virosomes primed for Th1-type responses as evidenced by high IFNγ levels and low IL5 levels, not only in *ex vivo* cultures of splenocytes from immunized mice stimulated with RSV antigen, but also in the lungs of immunized mice upon challenge with live RSV. Finally, mice vaccinated with RSV-MPLA virosomes were protected from challenge with live RSV without symptoms of ERD, as demonstrated by the absence of lung pathology and a lack of eosinophil infiltration into the lungs.

## Materials and Methods

### Ethical Statement

Animal experiments were evaluated and approved by the Committee for Animal Experimentation (DEC) of the University Medical Center Groningen, according to the guidelines provided by the Dutch Animal Protection Act (permit number DEC 5239A). Immunizations and challenges were conducted under isofluorane anesthesia, and every effort was made to minimize suffering.

### Virus and Cell Culture

RSV strain A2 (ATCC VR1540) was kindly donated by Mymetics BV (Leiden, The Netherlands). The virus was grown in roller bottles on HEp-2 cells (ATCC, CL-23, Wesel, Germany) in HEp-2 medium: DMEM (Invitrogen, Breda, The Netherlands) supplemented with Pen/Strep, L-Glutamine, Sodium bicarbonate, HEPES, Sodium Pyruvate, 1X non-essential Amino Acids (all from Invitrogen) and 10% FBS (Lonza-Biowhittaker, Basel, Switzerland) unless stated otherwise. At 80% CPE (5 days post-infection) the medium was cleared by low-speed centrifugation. Aliquots of the supernatant were snap-frozen in liquid nitrogen, as a source of live virus for immunization and challenge. The remainder of the virus was pelleted by ultracentrifugation and subsequently purified on a sucrose gradient. Purified virus was snap-frozen in liquid nitrogen and stored at −80°C in 20% sucrose in HNE buffer (5 mM Hepes, 145 mM NaCl, 1 mM EDTA, pH 7.4).

Mouse dendritic cells (DCs) were derived from bone-marrow cultures, as described before [33]. Briefly, both tibia and femurs were flushed with Iscove’s modified DMEM (IMDM; Invitrogen,) supplemented with 10% FBS, pen/strep, 0.1% *β*-mercaptoethanol (Invitrogen). Red blood cells were lysed by incubating the cells with ACK buffer (0.83% NH_4_Cl, 10 mM KHCO_3_, 0.1 mM EDTA, pH 7.2) for 5 min on ice. The cells were washed with IMDM medium and incubated in IMDM medium supplemented with 200 ng/ml Fms-like tyrosine kinase 3 ligand (Flt3L)(R&D systems, Abingdon, UK). Medium was replaced after 4 days and dendritic cells were harvested 8 days after initiation of the culture.

HEK-Blue TLR4 and HEK-Blue Null2 cells were purchased from Invivogen (Toulouse, France) and maintained according to the manufacturer’s protocol.

### Vaccine Production

RSV virosomes were generated as described previously [21]. Briefly, purified RSV was pelleted by ultracentrifugation and dissolved in 100 mM 1,2 dihexanoyl-*sn-*glycero-3-phosphocholine (DCPC) in HNE buffer. The nucleocapsid was removed by ultracentrifugation. Subsequently, a 2∶1 molar mixture of egg phosphatidylcholine (PC) and egg phosphatidylethanolamine (PE) (Avanti Polar Lipids, Alabaster, AL, USA) in 2∶1 chloroform/methanol at 850 nmol/mg protein was evaporated to a dry film in a glass tube. The supernatant containing the membrane lipids and proteins was added to the lipid mixture. For incorporation of MPLA, monophosphoryl lipid A from *Salmonella minnesota* Re 595 (Invivogen) was first dissolved in 100 mM DCPC in HNE buffer and then added to the protein/lipid mixture at 1 mg MPLA/mg virosomal protein. For the MPLA concentration experiment, MPLA was added in lower ratios i.e. 1∶0.2, 1∶0.04, 1∶0.008 (mg virosomal protein to mg MPLA). The mixture was incubated for 15 min at 4°C, filtered through a 0.22 µm filter and dialyzed in a sterile Slide-A-lyzer (10 kD cut-off; Thermo Scientific, Geel, Belgium) against 4×2 liters of HNE pH 7.4 for 48 hours. After dialysis, virosomes were kept at 4°C.

FI-RSV vaccine was produced according to the original protocol, which was used for the 1960’s FI-RSV preparation as reported in [34]. FI-RSV was diluted in HNE buffer to contain 5 µg of RSV protein in 25 µl of vaccine.

### *In vitro* Analyses

The virosomes were analyzed by equilibrium density gradient centrifugation on 10–60% sucrose gradients in HNE. Gradients were spun for 60 hr in an SW 55 Ti rotor at 50000 rpm and samples from the gradient were analyzed for protein, phospholipid phosphate and density (by refractometry). Each fraction was dialyzed against HNE in a Slide-A-Lyzer MINI Dialysis Device (Thermo Scientific, Geel, Belgium) overnight to remove the sucrose which is toxic for HEK-Blue cells at high concentrations. The samples were corrected for increases in volume due to the dialysis and 20 µl volumes of the samples were used to stimulate HEK-Blue TLR4 cells (10^5^ cells/well) and HEK blue Null2 cells (5×10^4^ cells per well) overnight at 37°C in a 96 well plate in triplicate. To quantify alkaline phosphatase production, 20 µl of HEK-Blue cell supernatant was added to 180 µl Quanti-Blue (Invivogen, Toulouse, France) and incubated for 30 minutes at 37°C. Absorbance was measured at 630 nm and plotted relative to the activation induced by 100 ng/ml of TNFα.

Upregulation of surface markers was assessed after incubating DCs with different virosome preparations. DCs were incubated at 1×10^6^ cells/ml at 37°C in IMDM medium. The incubation was stopped after 24 hr by washing the cells twice in medium. Expression of surface markers was determined by staining with anti-mouse CD80-PE (12-0801-82, eBioscience, Vienna, Austria) anti-mouse CD86- PE (12-0862-82, eBioscience) and anti-mouse CD40-FITC (11-0402-82, eBioscience) using standard staining protocols, followed by flow-cytometric analysis on a FACSCalibur flow cytometer (BD Bio- sciences, Erembodegem, Belgium).

### Animal Experiments

Female specified-pathogen-free BALB/c OlaHsd mice (6–8 weeks old) (Harlan, Zeist, The Netherlands) were used for all immunization experiments. For immunization and challenge, mice were anesthetized using 3–4.5% isoflurane in O_2_. Mice received RSV virosomes, RSV-MPLA virosomes or FI-RSV intramuscularly in 25 µl HNE. Each preparation contained 5 µg of protein. Control mice received 50 µl (1*10^6^ TCID_50_) of live RSV, intranasally or 25 µl of HNE intramuscularly. Vaccinations were given on day 0 and day 14. On day 28 mice were challenged with 10^6^ TCID_50_ (titrated as described below) of live RSV intranasally. On time points of vaccination and challenge, blood was drawn by retro-orbital puncture. Four days after challenge, mice were sacrificed and blood was drawn by heart puncture. Spleens were harvested for analysis of RSV-specific T cell cytokine responses and lungs for analysis of pathology, determination of lung cytokines and viral titers, respectively.

### Virus Titration

Virus titers were determined by titration of the tissue-culture infectious dose (TCID_50_). For challenge virus, initial dilutions of 1∶5000 were made in HEp-2 medium without FBS. Serial twofold dilutions of these samples were made in 96-well plates in quadruplicate. 20,000 HEp-2 cells were added to the virus dilutions and incubated for 5 days at 37°C in 5% CO_2_. The cells were then fixed with 1% paraformaldehyde in PBS for 45 min, blocked with 2% milk powder (Protifar plus, Nutricia, Zoetermeer, The Netherlands) in PBS for 1 hr and stained with 50 µl 1∶400 FITC-labeled goat anti-RSV antibody (Meridian life science Inc, Saco, ME, USA) at 37°C overnight. The next day, plates were washed with PBS and analyzed under a fluorescence microscope. Wells were considered positive for infection if one or more fluorescent syncytium was present. Titers were calculated using the Reed & Muench method.

To determine virus titers in the lungs of challenged mice, the lungs were removed aseptically after euthanasia of the mice. Lungs were then homogenized in 1 ml of 2% FBS containing HEp-2 medium using an automated Potter homogenizer Polytron-Aggregate® (Thomas Scientific, Swedesboro, NJ, USA). Next, homogenates were centrifuged at 1400 rpm for 10 min at 4°C, and supernatants, diluted to a 1∶5 starting dilution, were used to determine viral titers using the TCID_50_ method as described above.

### *In vitro* Neutralization Assay

Volumes of 100 µl of serum were heat-inactivated for 30 min at 56°C and subsequently diluted with 150 µl serum-free HEp2 medium. Wells of 96-well plates were filled with 50 µl of serum free HEp2 medium. Fifty µl of diluted serum was applied to the first row of wells in quadruplicate and serial two-fold dilutions were made. Subsequently, 70 TCID_50_ of live RSV was added in 50 µl of serum free HEp2 medium and incubated at 37°C for 2 hr. After incubation, 20,000 HEp2 cells were added per well in 100 µl of HEp2 medium with 4% FBS. After 5 days of incubation, the cells were washed, fixed and stained as described above for the virus titration. Neutralization titer was calculated with the Reed & Muench method and is indicated as the reciprocal of the dilution that neutralizes infection in 50% of the wells.

### Immunological Assays

RSV-specific antibody titers were determined as described before [21]. Briefly, 96-well plates were coated with betapropiolactone-inactivated RSV and then blocked with 2.5% milk powder in coating buffer. Plates were then incubated for 90 min with two-fold serial dilutions of serum or broncho-alveolar lavages (BAL; see below), starting at 1∶200 for serum or 1∶1 for BAL. After washing, plates were incubated with a 1∶5000 dilution of horseradish-peroxidase-coupled goat anti-mouse IgG, IgG1, IgG2a, IgA, or rat anti-mouse IgE (Southern Biotech 1030-05, 1070-05, 1080-05, 1040-05, 1130-05) for 1 hr, washed again and subsequently stained with *o*-Phenylenediamine (OPD; Sigma-Aldrich, St Louis, MO, USA). After 30 min the staining was stopped by addition of 2 M H_2_SO_4_ and absorption was measured at 492 nm. For levels of total IgG, geometric mean titers (GMT) were determined. For quantification of IgG1 and IgG2a levels, a calibration curve was used. For this, ELISA plates were coated with goat anti-mouse-IgG (heavy and light chain, human absorbed; Southern Biotech, 1031-01) at 100 ng/well in coating buffer overnight at 37°C. After blocking with 2.5% milk powder, known concentrations of a mouse IgG1 isotype control (Southern Biotech, 1070-01) and mouse IgG2a isotype control (Southern Biotech, 0103-01) were prepared, and applied to the plates. After a 90-min incubation at 37°C, plates were washed and stained as described above.

For analysis of levels of IL5 and IFNγ in splenocyte cultures and lung homogenates (see below), mouse IFN-γ and mouse IL5 high sensitivity ELISA kits (eBioscience) were used according to the manufacturer’s instruction.

For the analysis of IFN-γ and IL5 secretion in the RSV-specific recall responses of splenocytes, spleens were removed four days after challenge and transferred to a 15 ml tube containing IMDM/10% FCS. The spleens were passed through a 70-µm cell strainer (BD Biosciences, Heidelberg, Germany) using sterile 3-mL syringe plungers. Erythrocytes were then lysed by incubating with ACK buffer for 5 min on ice. The cells were washed with medium, counted and seeded at 2×10^6^ cells/ml and stimulated with BPL-RSV (10 µg/mL) in IMDM/10% FCS in triplicates and incubated at 37°C in a 5% CO_2_ atmosphere for 72 hr. Supernatants were harvested and stored at −20°C until further analysis.

For analysis of IL5 and IFNγ levels in RSV-infected lungs, lungs were removed from challenged mice and homogenized using the method as described for virus titration (see above). IL5 and IFNγ levels were then determined in supernatants of centrifuged lung homogenates.

### Lung Histopathology

The harvested lung lobes were inflated with 4% formalin in PBS and subsequently embedded in paraffin. Four µm slices were then prepared, and stained with standard hematoxylin and eosin. After staining, lung inflammatory parameters (peribronchiolitis, perivaculitis and alveolitis) were assessed by light microscopic analysis of slides.

### Broncho-alveolar Lavage Cytospins

BAL were taken by rinsing the lungs of the mice with 1 ml of PBS supplemented with protease inhibitors using a winged shielded i.v. catheter (1.3×30 mm, BD Utah) inserted, through an incision, in the trachea of euthanized mice. Cells in the BAL were pelleted by low-speed centrifugation and resuspended in 500 µl PBS. In some cases, the remaining BAL supernatants were used for IgA antibody assessment in ELISA. Subsequently, cells were spotted (300 rpm for 5 min) onto glass slides, air dried, and fixed in 80% methanol/20% PBS (V/V) for 10 min at −20°C. After air-drying, slides were stained for 20 min in May-Grunwald-Giemsa stain (Merck, Darmstadt, Germany), diluted 1∶1 in Sørensen’s phosphate buffer (0.2 M; pH 6.6). Then, slides were rinsed in Sørensen’s phosphate buffer, and incubated for 15 min in Giemsa stain (Merck, Darmstadt, Germany) diluted 1∶8 in Sørensen’s phosphate buffer. After washing with tap water, slides were air-dried and spots were sealed using cover slides and Kaiser’s glycerol (Merck, Darmstadt, Germany). The presence of eosinophils in cytospot BAL cells was analyzed by light microscopy.

### Statistical Analysis

All statistical analyses were performed with Graphad Prism 5.00 for Mac OSX, (GraphPad Software, San Diego California USA, www.graphpad.com. Statistical significance was assessed using a Mann-Whitney U test. A P value of 0.05 or lower was considered to represent a significant difference.

## Results

### Characterization of RSV-MPLA Virosomes

**Figure 1:**
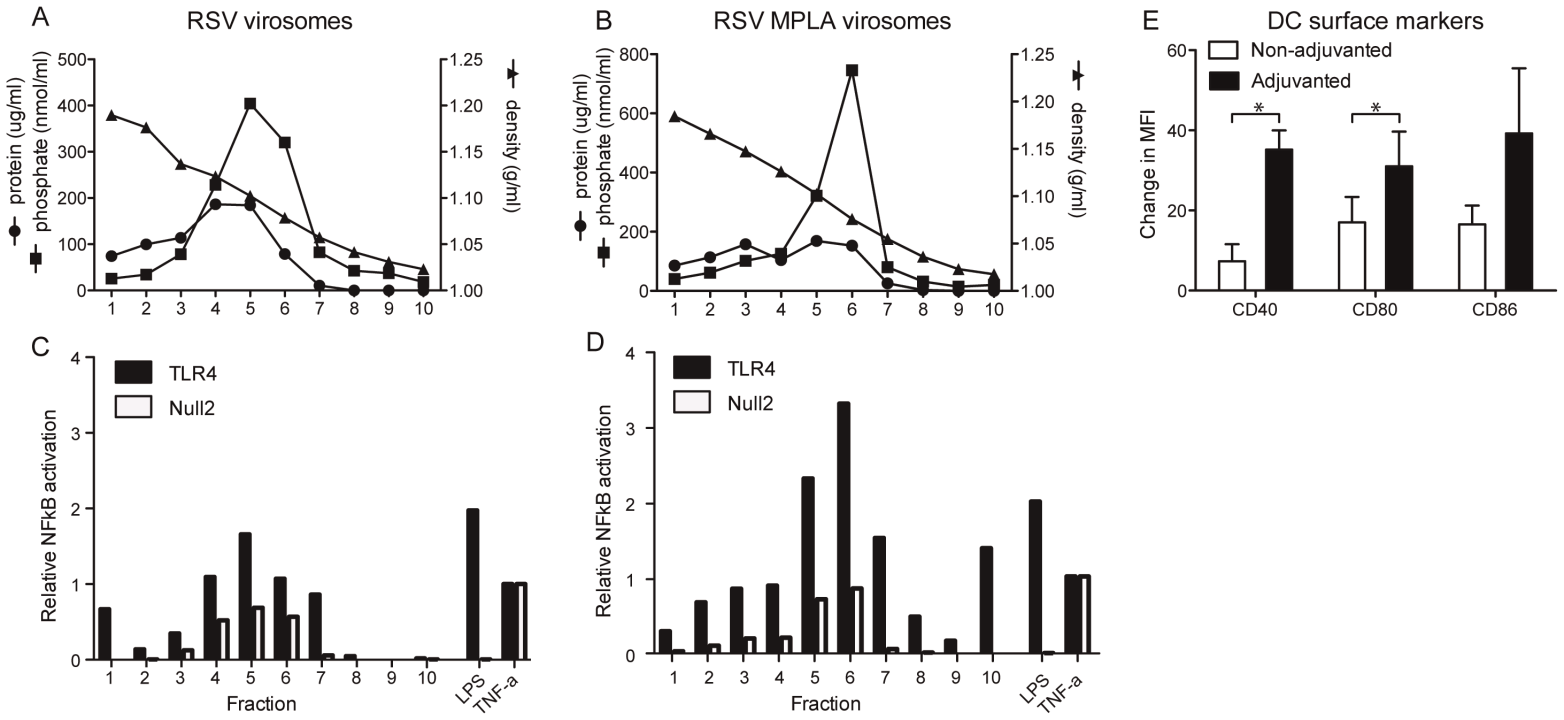
*In vitro* analysis of RSV and RSV-MPLA virosomes. (A,B) RSV virosomes and RSV-MPLA virosomes were spun on an equilibrium density sucrose gradient. Subsequently, density, protein concentration, and phosphate concentrations of each fraction was determined. (C,D) Fractions from A and B were analyzed for their TLR4-signaling ability using Hek-Blue TLR4 cells. To assess non-TLR specific activation of cells, control cells (Null2 cells) were incubated with the same virosome fractions. As a control for activation both Hek blue TLR4 and Hek blue null2 cells were stimulated with 100 ng/ml TNF-α. Bars represent TLR activation relative to that of the TNF-α control (E) Upregulation of DCs costimulatory molecules CD40, CD86, CD80. Unfractionated virosome preparations were used to stimulate *ex vivo* cultured mouse DCs overnight. Cells were stained for expression of costimulatory molecules using specific monoclonal antibodies and analyzed by FACS. Bars represent the percentage of positive cells. The data shown are a representative of three individual experiments.

The formation of virosomes was analyzed by equilibrium density-gradient centrifugation. Protein and phosphate were found to co-migrate for RSV virosome preparations with and without MPLA, indicating successful reconstitution of the viral envelopes (Figure 1A, 1B). For RSV-MPLA virosomes, the apparent absence of phosphate outside the virosome peak indicated that MPLA was primarily associated with the virosomal membranes.

### *In vitro* Analysis of RSV-MPLA Virosomes

To assess the immune-potentiating capacity of the RSV-MPLA virosomes, fractions from the sucrose gradient were tested for their TLR4-activating activity in HEK-Blue TLR4 cells, after dialysis to remove the sucrose. The fractions containing the non-adjuvanted virosomes induced a TLR4-mediated NF-κB activation which was slightly higher than the activation induced by TNF-α (Figure 1C). This activation is probably due to TLR signaling of the RSV F protein [35]. Incorporation of MPLA into the virosomes strongly stimulated TLR4 signaling by the virosomes. The fraction at the top of the gradient also induced activation of TLR4, indicating that not all the added MPLA had been inserted in to the viral envelopes (Figure 1D). Since a large proportion of the MPLA was associated with the virosomal fraction, as judged by phosphate analysis and TLR4-activating capacity of the fractions of the gradient, subsequent experiments were performed with non-fractionated virosomes.

Next, virosomes were tested for their capacity to up-regulate costimulatory molecules in mouse DCs. Non-adjuvanted virosomes induced the upregulation of DC maturation markers CD40, CD80 and CD86. Incorporation of MPLA in to these virosomes significantly stimulated the induction of CD40 and CD80 expression compared to the induction by RSV virosomes (Figure 1 E).

### *In vivo* Immunogenicity

**Figure 2:**
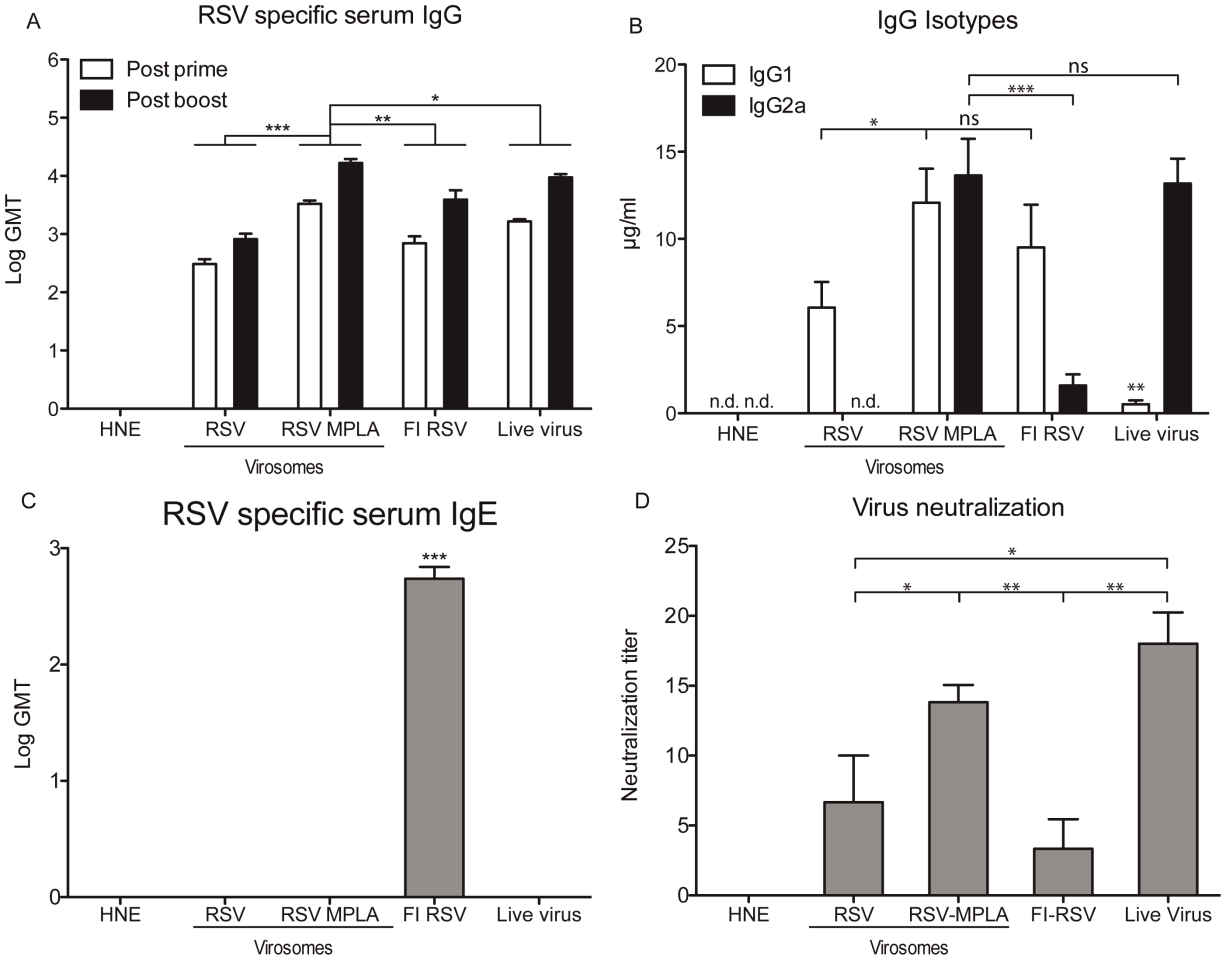
RSV specific IgG in mice after vaccination with RSV virosomes and RSV-MPLA virosomes. Mice were vaccinated twice with RSV virosomes, RSV-MPLA virosomes or controls (HNE, live virus and FI-RSV). Each injection contained 5 µg of protein. (A) RSV-specific IgG titers in serum 14 days after prime and 14 days after booster vaccination. (B) RSV-specific IgG1 and IgG2a subtype levels in serum 14 days after booster vaccination. (C) IgE levels were determined at 14 days after booster vaccination. (D) RSV neutralizing antibody titers in serum obtained 5 days after challenge. Bars represent the GMT (panels A and C), mean concentration of RSV-specific IgG1/2a (panel B) or mean neutralization titer (panel D) of 6 mice per group. Error bars represent the SEM. Statistical differences were calculated using the Mann-Whitney-U test. *p<0.05, **p<0.01, ***p<0.001. Statistical differences in IgE levels were calculated with an ANOVA with Bonferroni correction for multiple testing ***p<0.001. The data shown are a representative of two individual experiments.

To analyze the immunogenicity of the virosomes *in vivo*, Balb/c mice were vaccinated twice with RSV virosomes or RSV-MPLA virosomes at a 2-week interval. For comparison, mice were inoculated with live RSV (to induce a Th1-skewed immune response) or vaccinated twice with FI-RSV (to induce a Th2-skewed immune response). Two weeks after the first and second vaccination, blood was drawn and serum IgG titers were determined. After the priming immunization, RSV virosomes induced a mean IgG titer of 2.5 Log GMT. Incorporation of MPLA in to the virosomes resulted in significantly increased IgG levels after both priming and booster immunizations, not only compared to the levels induced by non-adjuvanted RSV virosomes but also to the levels induced by FI-RSV and live virus (Figure 2A).

Next, RSV-specific IgG1 and IgG2a subtype levels were determined. RSV-MPLA virosomes induced significantly higher levels of IgG2a compared to non-adjuvanted virosomes, reaching similar levels of RSV-specific IgG2a as seen after live virus inoculation (Figure 2B). In parallel with the increased RSV-specific IgG2a responses, increases in RSV-specific IgG1 levels were also noted. Non-adjuvanted RSV virosomes and FI-RSV mainly induced IgG1, indicative of a Th2-type response. Live virus inoculations induced low levels of IgG1 and similar levels of IgG2a, compared to those induced by RSV-MPLA virosomes (Figure 2B).

To further characterize the humoral immune response, we determined IgE levels in sera and IgA levels in BAL of immunized mice. IgE was exclusively induced by immunization with FI-RSV, but not by immunization with virosomes or live virus (Figure 2C). IgA in BAL was detectable in mice immunized with FI-RSV (4.6±0.1 ^2^Log GMT) and live virus (5.6±0.6 ^2^Log GMT), but not in mice immunized with virosomes. For assessment of the functional capacity of the antibodies, we performed a microneutralization assay. Non-adjuvanted RSV virosomes induced similar neutralizing antibody titers to FI-RSV. Incorporation of MPLA in to the virosomes significantly increased the neutralizing antibody titers to levels similar to those induced by live virus (Figure 2D).

**Figure 3:**
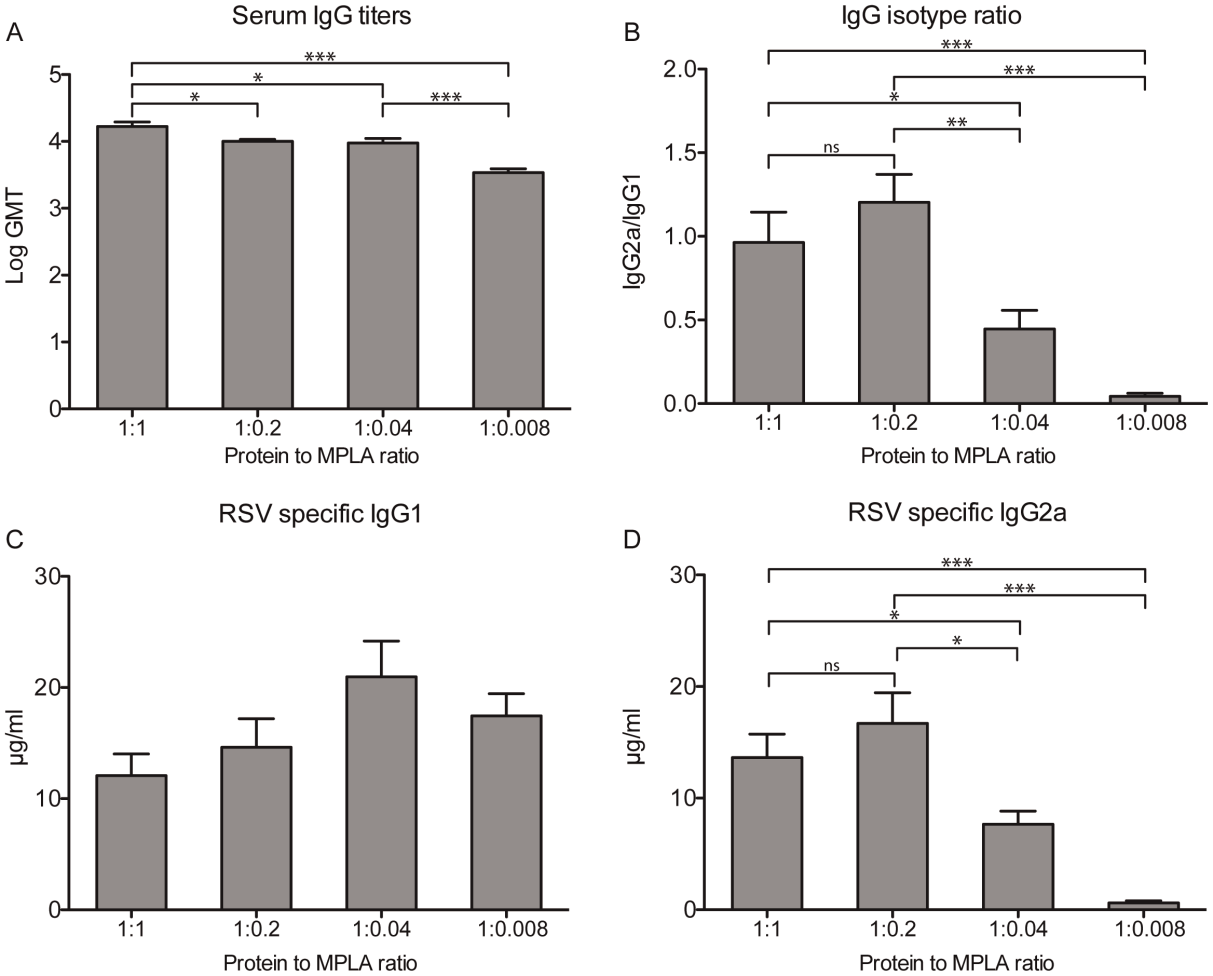
Influence of MPLA to virosome protein ratios on RSV specific IgG titers. Mice were vaccinated twice with RSV-MPLA virosomes (5 µg of protein) with different amounts of incorporated MPLA. 14 days after the second vaccination RSV-specific IgG titers in serum were determined. (A) RSV-specific IgG titers. (B) Ratio’s of RSV-specific IgG2a/IgG1 concentrations determined 14 days after booster vaccination. (C) RSV-specific IgG1 concentrations. (D) RSV specific IgG2a concentrations. Bars represent the GMT (panel A), mean ratio (panel B) or mean concentration of IgG1/2a of 6 mice per group. Error bars represent the SEM. Statistical differences were calculated using the Mann-Whitney-U test. *p<0.05, **p<0.01, ***p<0.001. The data shown are a representative of two individual experiments.

To investigate which concentration of MPLA is needed for optimal adjuvant activity, we added different amounts of MPLA to the viral protein in solution before reconstitution. Apart from the 1∶1 protein:MPLA ratio, we also produced virosomes with 1∶0.2, 1∶0.04 and 1∶0.008 protein to MPLA ratios. Using a similar immunization regimen and antigen dose as before, mice were vaccinated, and RSV-specific serum IgG and subtype responses were determined. The reduction in total RSV-specific serum IgG induced by the vaccine was proportional to the decline in the amount of MPLA in the virosomes (Figure 3A). The IgG2a/IgG1 subtype ratio remained similar when the amount of MPLA was reduced from 1 to 0.2 mg/mg protein but decreased when the amount of MPLA was reduced further (Figure 3B). This decrease was primarily due to a reduction in RSV-specific IgG2a levels, while the level of RSV-specific IgG1 did not increase significantly with lower amounts of virosome-incorporated MPLA (Figure 3C, 3D). Because there was no significant difference between the IgG subtypes induced by 1∶1 and 1∶0.2 protein to MPLA ratio virosomes and there are other benefits to be expected from higher MPLA concentrations (i.e. cellular immune response and reduction in lung pathology) we chose to perform the next experiments with 1∶1 protein:MPLA virosomes.

### Cellular Immunity

**Figure 4:**
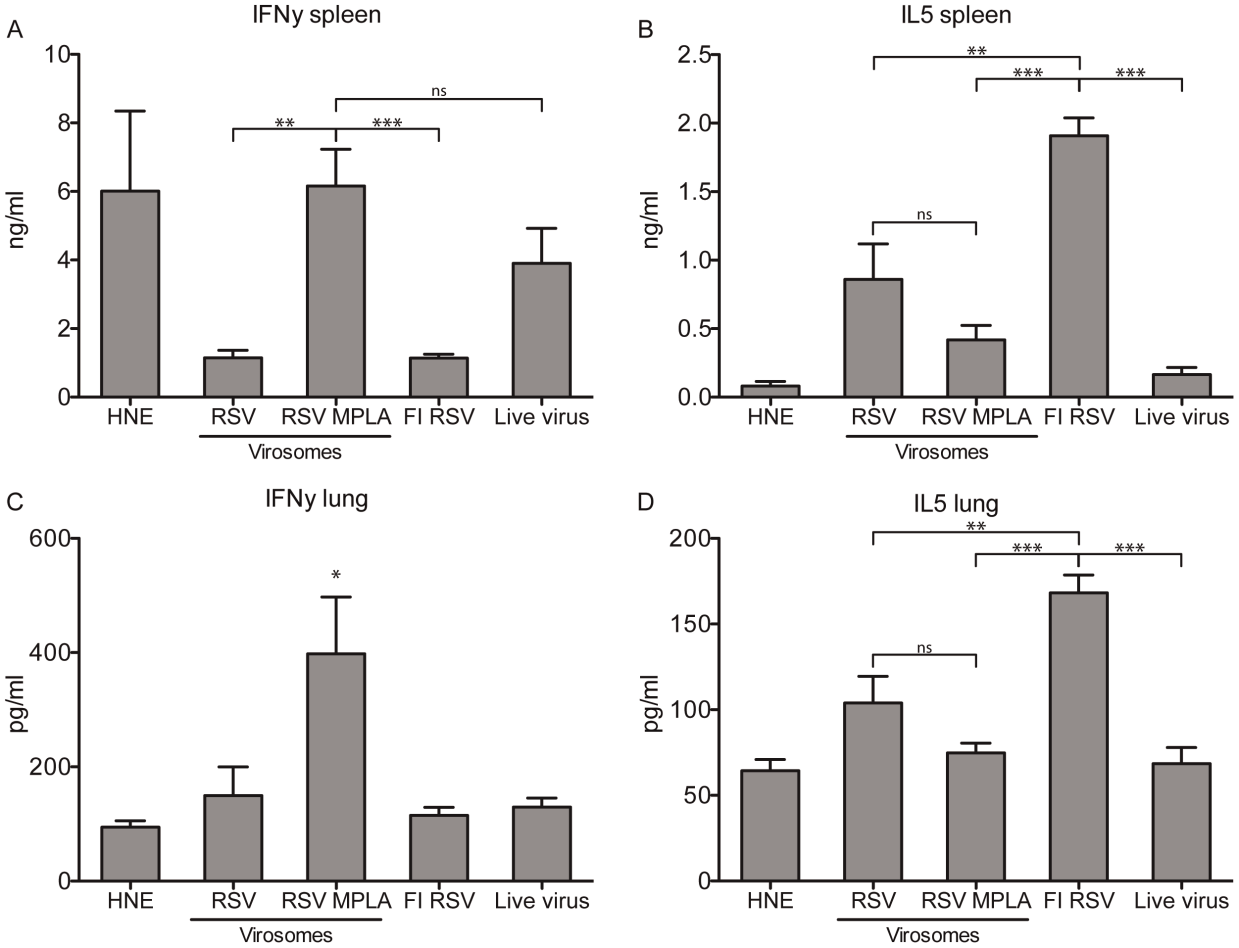
IFNγ and IL5 concentrations in RSV-stimulated splenocyte cultures and lung tissue homogenates. Mice were vaccinated twice with RSV virosomes, RSV-MPLA virosomes and control vaccines as in Figure 2, and subsequently challenged with live RSV. Four days after challenge, IFNγ and IL5 responses were determined. (A) IFNγ concentrations in splenocyte cultures restimulated with BPL-inactivated RSV for three days. (B) IFNγ concentrations in homogenated lung tissue, four days after challenge. (C) IL5 concentrations in splenocyte cultures, restimulated with BPL-inactivated RSV for three days. (D) IL5 concentrations in homogenated lung tissue, four days after challenge. Bars represent the mean cytokine concentration of 6 mice per group and error bars represent the SEM. Statistical differences were calculated using a Mann-Whitney-U test. *p<0.05, **p<0.01, ***p<0.001. The data shown are a representative of two individual experiments.

To analyze if virosome-incorporated MPLA skews the immune response to a favorable Th1 phenotype, levels of the hallmark Th1 cytokine IFNγ and Th2 cytokine IL5 were determined in splenocyte cultures of mice, *ex vivo* stimulated with RSV. Supernatants of splenocytes cultures from mice immunized with RSV-MPLA virosomes or infected with live virus produced significantly increased levels of IFNγ compared to those from mice immunized with RSV virosomes alone or FI-RSV (Figure 4A) Restimulated splenocytes from non-vaccinated mice produced considerable levels of IFNγ, which may be explained by activation of innate immunity (i.e. NK cell activation) as a result of a high viral load occurring in infected naïve animals. Levels of IL5 were significantly increased in splenocyte cultures from mice immunized with FI-RSV when compared to those from all other groups (Figure 4B).

Next, secretion of these cytokines was measured locally, *i.e.* in lung homogenates, 4 days after viral challenge. In line with the above data, mice immunized with RSV-MPLA virosomes showed significantly increased IFNγ levels in their lungs upon live virus challenge when compared to levels measured in the lungs of mice immunized with non-adjuvanted virosomes, FI-RSV or live virus immunization (Figure 4C). Also, IL5 levels were significantly increased in the lungs of FI-RSV immunized mice when compared to the levels measured in the lungs of mice immunized with (adjuvanted) RSV virosomes or live virus (Figure 4D).

### Virus Clearance after Challenge

**Figure 5:**
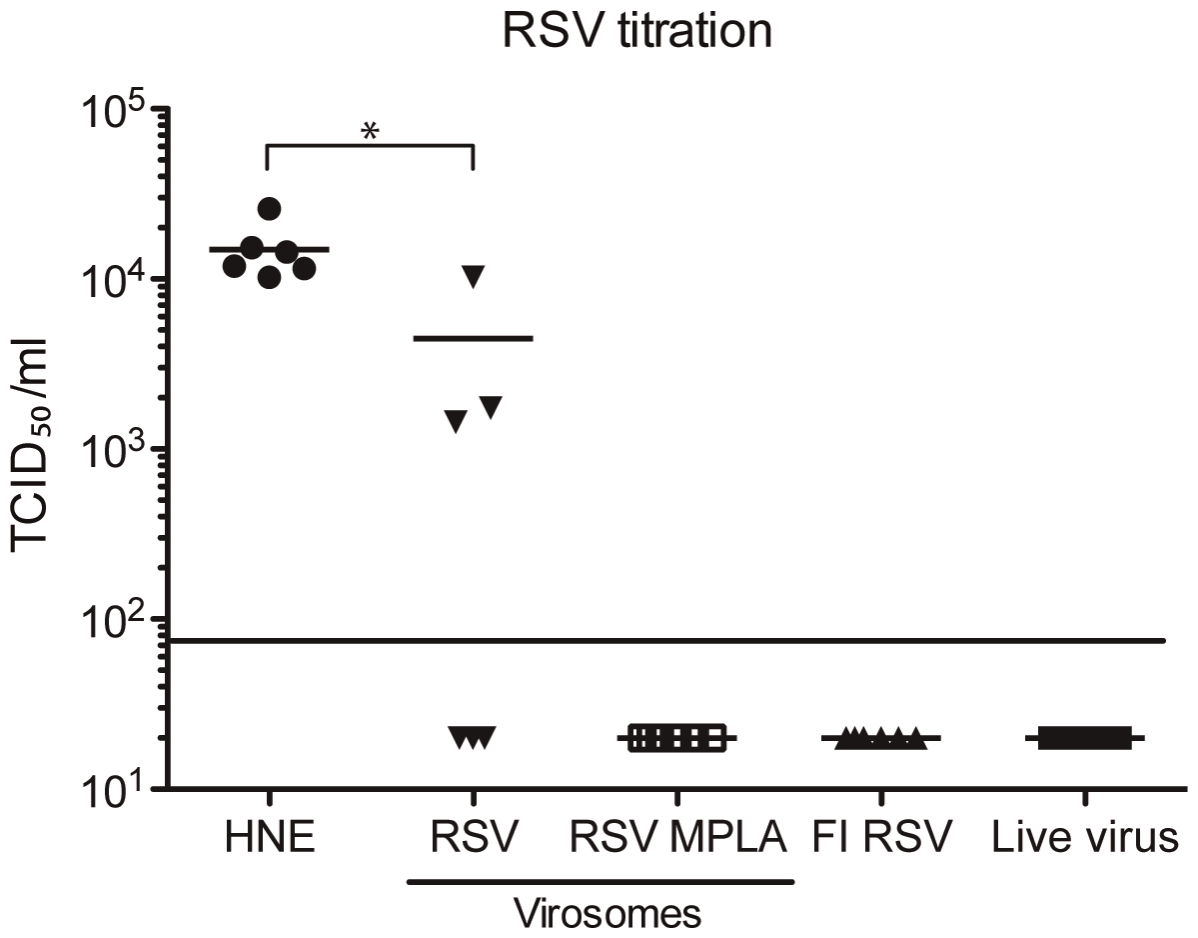
Protection against live virus challenge and infiltration of eosinophils. Mice were vaccinated as described in figure 2 and challenged with live virus 14 days after the booster vaccination. Four days after challenge, lungs were removed and the viral titer was determined and expressed as TCID_50_. RSV TCID_50_ titers from the lungs of challenged animals. Statistical differences were calculated using the Mann-Whitney-U test. *p<0.05. The data shown are a representative of two individual experiments.

To analyze vaccination-induced virus clearance after challenge, mice were immunized twice with HNE buffer, FI-RSV, live virus, RSV virosomes or RSV-MPLA virosomes. Two weeks after the second vaccination mice were challenged with 106 TCID_50_ live RSV. Four days later, viral titers were determined in the lungs of the animals. In the HNE vaccinated group, virus was recovered from the lungs of all mice (Figure 5A). In three out of the six mice immunized with RSV virosomes, virus could not be detected. In the other mice, virus was detected albeit at a significant lower level than in non-immunized mice. In contrast, in all mice immunized with RSV-MPLA virosomes, FI-RSV and live virus, virus could not be detected.

### Lung Pathology

**Figure 6:**
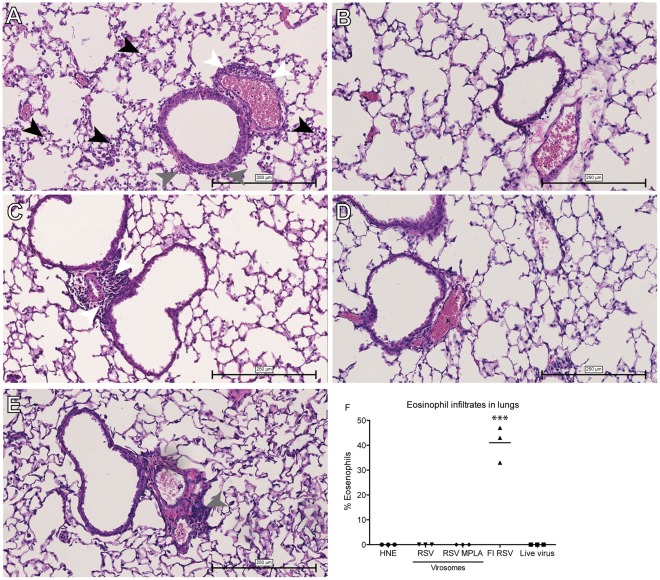
Lung pathology in mice after immunization and RSV infection. Mice were immunized and challenged as described in Figure 2 and the lungs were harvested, sliced and stained with H&E and assessed for pathology using light microscopy. Panels represent the lungs of (A) FI-RSV, (B) live virus, (C) RSV virosomes, (D) RSV MPLA virosomes (E) buffer immunized mice. Black arrows point to alveolar infiltrates, grey arrows to peribronchial infiltrates and white arrows to perivascular infiltrates. (F) Eosinophils in BAL expressed as percentage of total BAL cells. Data points represent values from individual mice. Statistical differences were calculated using the ANOVA test with Bonferroni correction for multiple testing. ***p<0.001. The data shown are a representative of two individual experiments.

To further investigate ERD in the immunized mice, we examined lung pathology upon challenge infection (Figure 6). Mice immunized with FI-RSV showed signs of alveolitis and infiltrates in both the peribronchial and perivascular areas (Figure 6A). The lungs of mice immunized with live virus on the other hand showed no signs of pathology (Figure 6B). Mice immunized with RSV virosomes showed no signs of alveolitis but did have perivascular infiltrates (Figure 6C) In contrast, the lungs of the mice who received RSV-MPLA virosomes showed no signs of lung pathology (Figure 6D) and were very similar to the lungs of mice who received live virus or those of non-immunized mice (Figure 6B,E). In addition to this, we assessed the presence of eosinophils in broncho-alveolar lavages (BAL) four days after challenge by May-Grunwald Giemsa staining of cytospotted cells. No eosinophils were detected in BAL of mice vaccinated with RSV or RSV-MPLA virosomes. On the other hand, in the mice vaccinated with FI-RSV, eosinophils were clearly present (Figure 6F).

## Discussion

Despite the fact that RSV has been recognized as an important vaccine target for more than 60 years, no vaccine is registered for use in humans today. Various vaccine candidates have been evaluated in clinical trials but so far none of them showed the required safety and efficacy profiles. Generally, live attenuated virus vaccines administered intranasally are safe and well tolerated but it is difficult to obtain an optimal balance between immunogenicity and attenuation [36]. Inactivated virus vaccines appear to be hard to advance to the clinic because of the safety concerns related to the outcome of the 1960’s FI-RSV trial. Protein subunit vaccines are easy to produce but are generally not very immunogenic and possibly skew towards a Th2 immune response [36].

In this study, we evaluated the immunogenicity and protective capacity of a virosomal RSV vaccine adjuvanted with MPLA. Incorporation of the TLR4 ligand MPLA into the virosomal membrane resulted in effective human TLR4 stimulation in HEK-Blue cells *in vitro* and activation of mouse DC *ex vivo* as shown by the upregulation of co-stimulatory molecules. Incorporation of MPLA in virosomes resulted in increased RSV-specific serum IgG titers, with production of RSV-specific, Th1-signature, IgG2a-isotype antibodies similar to that induced by live virus inoculation leading to a balanced IgG1/IgG2a profile. These antibodies proved effective in virus neutralization. Furthermore, RSV-MPLA virosomes skewed the cellular responses towards a Th1 profile, as shown by enhanced IFN-γ secretion, not only in *ex vivo* RSV-stimulated splenocytes, but also locally in the lungs of infected mice. Immunization with RSV-MPLA virosomes did not induce any detectable IgE in contrast to immunization with FI-RSV. IgE induction is a hallmark of a Th2-skewed allergy-like response, which is implicated in RSV infections and in FI-RSV induced enhanced disease [37]–[39]. MPLA-adjuvanted virosomes, similar to FI-RSV, provided full protection against live RSV infection, but in contrast to FI-RSV, did not lead to signs of ERD, *i.e.* influx of eosinophils in the lungs or induction of lung pathology. Importantly, previous studies in cotton rats showed that addition of MPLA to FI-RSV reduces the induction of ERD by FI-RSV immunization, illustrated by a reduction in lung pathology, an increase in serum virus neutralization titers and a shift from a Th2 -skewed immune response to a balanced immune response [31], [32]. Our observations on the immune response induced by MPLA-adjuvanted RSV virosomes in mice are in line with these data and underline that MPLA-adjuvanted RSV virosomes hold promise as a candidate RSV vaccine. Currently, RSV-MPLA virosomes are being evaluated in cotton rats to optimally assess other ERD parameters, such as alveolitis, in more detail.

Our data show that non-adjuvanted RSV virosomes stimulate human TLR4 in HEK-Blue cells and upregulate co-stimulatory molecules in mouse DC and that incorporated MPLA further enhances these effects. TLR4 activation by RSV virosomes without MPLA is likely to be caused by the RSV F protein. RSV F is a known TLR4 agonist that, for example, induces inflammatory cytokines like IL-6 in DC [35]. Interestingly, despite this capacity to stimulate TLR4, RSV virosomes fail to induce Th1-type responses while MPLA, also a TLR4 agonist, effectively stimulates Th1-type responses. This could be due to differences in the magnitude of stimulation, which is clearly higher for MPLA (Figure 1), but could also be caused by recruitment of different adaptor molecules downstream of TLR4 activation. As TLR4 uses both MyD88 and TRIF adaptor molecules, it is possible that MPLA competes with RSV F for TLR4 activation. This competition shifts signaling from RSV F-induced, MyD88-dependent, TLR4 signaling to MPLA-induced, TRIF-dependent, TLR4 signaling, leading to a Th1-skewed immune response induced by RSV-MPLA virosomes compared to non-adjuvanted RSV virosomes.

Apart from its influence on T helper cell differentiation, TLR signaling also has a direct effect on IgG isotype switching [40]. Antibody isotype switching is important, because different immunoglobulin subclasses display differences in their ability to mediate effector responses [41]. In mice, the most effective IgG isotype protecting against viral infections is IgG2a [42]. As stated before, MPLA signals through TLR4 to induce type I IFNs which stimulate IgG2a production predominantly from follicular B cells [40]. Furthermore, MPLA could also directly activate TLR4 on B cells to facilitate isotype switching, a process that is further augmented by IFNγ and T-cell help [43]. Previously, we incorporated TLR2 ligand P3CSK4 in RSV virosomes. P3CSK4 inclusion also skewed towards a Th1 immune response and increased IgG2a levels compared to non-adjuvanted virosomes. P3CSK4 adjuvanted RSV virosomes did however, induce slightly higher IgG1 than IgG2a levels. Incorporation of MPLA in the virosomes induces similar IgG1 and IgG2a levels. The relative increase of IgG2a levels compared to P3CSK4-RSV virosomes could be due to increased type I IFN production induced by MPLA. Since incorporation of MPLA in virosomes increases IgG2a levels compared to non-adjuvanted RSV virosomes or FI-RSV, antibodies induced by RSV-MPLA virosomes may well be more effective in protection against viral infection than antibodies induced by the non-adjuvanted RSV virosomes or FI-RSV.

Production of virosomes does not include the application of cross-linking chemicals for inactivation of the virus. This could well be a major advantage of the use of virosomes compared to other approaches using whole inactivated virus. In this respect, it is important to note that one of the reasons why FI-RSV failed to elicit virus-neutralizing antibodies is that important epitopes on the virus are disrupted by formalin [44]. One of the most important RSV epitopes for neutralizing antibodies is a specific conformational epitope making it very susceptible for alteration by chemical treatments, including inactivation with formalin [45]. During virosome production, inactivation occurs through disruption of the membrane by the short-chain phospholipid DCPC followed by removal of the nucleocapsid. This is then followed by reconstitution of RSV F and G protein in the viral membrane with retention of their native conformation. Following this procedure, RSV virosomes lack viral RNA and thus are fully replication-incompetent [21]. Preliminary data indicate that RSV virosomes expose all of the most important known protective on the RSV F protein, as demonstrated by efficient binding of monoclonal antibodies directed to these epitopes (unpublished results).

In conclusion, our data show the feasibility of producing RSV virosomes that have incorporated MPLA. MPLA improves the immunogenicity of RSV virosomes and skews immune response to a protective, balanced Th1/Th2-type response without priming for adverse immune reactions, such as eosinophil influx into the lung after infection with RSV. These data combined with the favorable safety profile of MPLA, and the fact that MPLA is already licensed for use in human vaccines, make the RSV-MPLA virosomal vaccine a suitable candidate for further evaluation in clinical trials.
